# Treatment decisions in a man with Hodgkin lymphoma and Guillian-Barré syndrome: a case report

**DOI:** 10.1186/1752-1947-8-455

**Published:** 2014-12-21

**Authors:** Caren L Hughes, Jeffrey T Yorio, Craig Kovitz, Yasuhiro Oki

**Affiliations:** 11 Division of Pharmacy, The University of Texas MD Anderson Cancer Center, 1515 Holcombe Boulevard, Houston, TX 77030 USA; 12 Division of Cancer Medicine, The University of Texas MD Anderson Cancer Center, 1515 Holcombe Boulevard, Houston, TX 77030 USA

**Keywords:** Guillian-Barre, Hodgkin lymphoma, Chemotherapy, Paraneoplastic

## Abstract

**Introduction:**

Guillain-Barre syndrome, or acute inflammatory demyelinating polyneuropathy, has been described in the presence of malignancies such as lymphoma. Guillain-Barre syndrome/acute inflammatory demyelinating polyneuropathy causes paresthesias and weakness, which can make the treatment of lymphoma with chemotherapy challenging. Given the rarity of this co-presentation it is not known if the effects of Guillain-Barre syndrome should be considered when selecting a treatment regimen for Hodgkin lymphoma. To the best of our knowledge, the impact of these treatment modifications has not been previously reported.

**Case presentation:**

We report the case of a 37-year-old Caucasian man with a diagnosis of stage IIB classical Hodgkin lymphoma with concomitant Guillain-Barre syndrome. Our patient originally presented with an enlarged cervical lymph node and quickly developed distal paresthesia and progressive weakness of all four extremities. He was diagnosed with Hodgkin’s lymphoma and initiated on treatment with doxorubicin, bleomycin, vinblastine, and dacarbazine. Doses of bleomycin and vinblastine were held or dose-reduced throughout his initial treatment course due to underlying neuropathy and dyspnea. He continued to have persistent disease after five cycles of doxorubicin, bleomycin, vinblastine, and dacarbazine and went on to receive salvage treatments including more chemotherapy, radiation, autologous stem cell transplant and is currently preparing for an allogeneic stem cell transplant.

**Conclusions:**

Paraneoplastic syndromes such as Guillain-Barre syndrome/acute inflammatory demyelinating polyneuropathy can make the treatment of patients with Hodgkin lymphoma more challenging and can interfere with delivering full-dose chemotherapy. Further case series are needed to evaluate the effect that paraneoplastic syndromes, or adjustments made in therapy due to these syndromes, negatively affect the prognosis of patients with Hodgkin lymphoma.

## Introduction

Paraneoplastic syndromes can develop in patients with cancer and indicate damage to distant organs or tissues via mechanisms external to the cancer itself [1, 2]. They are seen in less than 1% of patients with malignancies and cause a variety of disorders, including hematologic, dermatologic, renal and neurologic abnormalities [1, 2]. Guillain-Barré syndrome (GBS), or acute inflammatory demyelinating polyneuropathy (AIDP), is characterized by distal paresthesia and progressive bilateral, symmetric weakness of the extremities [3]. It is often precipitated by underlying infection, but has also been well described in the presence of malignancies, particularly lymphomas [4–10]. Since these associations are rare with only case reports in the literature, it is not truly known if the presence of paraneoplastic syndromes, such as GBS, should be considered when determining treatment intensity, or if the co-presence of these syndromes affects the prognosis of patients with underlying lymphoma. We describe the treatment and course of disease in a young man with Hodgkin lymphoma (HL) with associated GBS.

## Case presentation

A 37-year-old Caucasian man with a history of hypertension presented to his primary care physician with rhinorrhea, a cough and an enlarged lymph node in the left posterior neck that had been worsening over the past one to two months. Our patient was initially treated with a course of oral antibiotics, but had no improvement in his symptoms. He then developed intermittent numbness and tingling in his bilateral feet, which progressed to involve both of his legs and his fingertips. After a few more days, he developed weakness in his bilateral lower extremities and his hands. A computed tomography (CT) scan of his head revealed no significant abnormalities. A fine-needle aspiration of the neck lymph node was suggestive of, but not definitive for, HL. Excisional biopsy of a left supraclavicular lymph node was consistent with classical HL, nodular sclerosis-type.

Further workup with a positron emission tomography (PET)/CT scan revealed small volume left cervical, supraclavicular, axillary and mediastinal lymphadenopathy. A bone marrow biopsy demonstrated a cellular bone marrow with a slight increase in eosinophils, but no evidence of lymphoma, so our patient was given a diagnosis of stage IIB classical HL. By that time, the weakness in his arms and legs had become worse and our patient had experienced at least two episodes of falling with difficulty getting up without assistance. Our patient had right facial weakness, three out of five strength in his bilateral shoulders and hip flexors, four out of five strength in his knee flexors and decreased vibratory sensation up to the knees bilaterally. Patellar and Achilles reflexes were not present.

A magnetic resonance imaging (MRI) scan of his brain showed no intracranial abnormality. An MRI scan of his spine showed subtle nerve root enhancement of the cauda equine, but cerebrospinal fluid was negative for any evidence of malignant cells with only one white blood cell present (CSF protein = 205). Findings from an electromyelogram and nerve conduction study were consistent with an AIDP or GBS. Our patient received two days of intravenous immune globulin (IVIG) at a dose of 1g per kg with complete resolution of his sensory complaints and improvement in his weakness. Three days later, his neurologic symptoms had worsened along with development of some mild shortness of breath. Plasma exchange was initiated for a course of five days with resolution of his shortness of breath and mild improvement of his weakness and sensory deficit.

The following week, chemotherapy with doxorubicin, bleomycin and dacarbazine was initiated (Table 1). Vinblastine was originally omitted from the standard doxorubicin, bleomycin, vinblastine, and dacarbazine (ABVD) regimen to avoid exacerbation of our patient’s neuropathy. It was added back during cycle 2 and again held after our patient developed redness and tenderness in his fingertips following 3a. It was reintroduced at a reduced dose after cycle 4 and at full dose for cycle 5.

**Table 1:** **Chemotherapy doses, ABVD**

Infusion date cycle	Adriamycin mg/m ^2^	Bleomycin units/ m ^2^	Vinblastine mg/m ^2^	Dacarbazine mg/m ^2^
4/29/11	25	10	None	375
C1D1				
5/13/11	25	None	None	375
C1D15				
5/27/11	25	None	4	375
C2D1				
6/16/11	25	10	6	375
C2D15				
7/1/11	25	10	6	375
C3D1				
7/14/11	25	10	None	375
C3D15				
7/29/11	25	10	3	375
C4D1				
8/11/11	25	10	3	375
C4D15				
8/25/11	25	10	6	375
C5D1				
9/08/11	25	None	6	375
C5D15				

A, adriamycin; B, bleomycin; C, cycle; D, day; D, dacarbazine; V, vinblastine.

Bleomycin was omitted for cycle 1b and cycle 2a due to the development of cough and a 20% reduction in diffusion lung capacity for carbon monoxide (DLCO) on repeat pulmonary function tests (PFTs), but then reintroduced once our patient’s cough resolved. It was again omitted after cycle 5a due to worsening shortness of breath and further decrease in DLCO on PFTs.

By the start of cycle 2, our patient’s Eastern Cooperative Oncology Group (ECOG) performance status had improved from two to one and our patient no longer needed a cane for ambulation. Our patient was also treated with another dose of IVIG, which helped further improve his neurologic symptoms. After cycle 3, a PET/CT scan showed significant improvement in left neck, left axillary, left hilar and mediastinal lymph nodes. Since there was not complete resolution of the lymphadenopathy, our patient was continued on ABVD. A repeat PET/CT scan after cycle 5 showed residual disease with an increase in metabolic activity, so a biopsy was performed of a left neck lymph node and revealed persistent classical Hodgkin lymphoma.

Our patient was then treated with ifosfamide, carboplatin, etoposide (ICE) for two cycles with persistent disease, gemcitabine and vinorelbine for two cycles with persistent disease followed by brentuximab vendotin for three cycles with progressive disease. Note that brentuximab can cause peripheral sensory neuropathy; our patient experienced numbness and tingling in the lower extremities that resolved after four days.

Since our patient still only had localized disease at that point, intensity-modulated radiation therapy (IMRT) was given to the mediastinum, neck and axilla at a total dose of 30.6Gray (Gy) in 17 fractions with an additional 5.4Gy boost to the mediastinum and neck. He then underwent an autologous stem cell transplant with carmustine, etoposide, cytarabine and melphalan conditioning.

Our patient went into remission for about six months, but then developed recurrent disease in his right lower lung. He is currently undergoing chemotherapy with bendamustine with plans for an allogeneic stem cell transplant. Interestingly, his neurologic symptoms had mostly resolved despite the persistence of his HL.

## Discussion

This report describes a young man with simultaneous diagnoses of HL and GBS. Lymphocytes are primary drivers of the immune process, and dysregulation of the immune system can be one outcome of lymphocytic malignancies [11]. In one study of 519 patients with HL, 9% of patients were diagnosed with a lymphoma-associated autoimmune disease. These autoimmune processes are the underlying etiology of many of the paraneoplastic disorders reported for patients with lymphoma [2]. The abnormalities can be hematologic (autoimmune hemolytic anemias, immune thrombocytopenic purpura, blood eosinophelia), dermatologic (Sweet’s syndrome, eosinophilic fasciitis), renal (glomerulonephritis) or neurologic.

Neurologic effects experienced by a patient with HL can be the result of the disease itself (infiltration of nerves by tumor, more likely in non-Hodgkin lymphoma (NHL) than HL), chemotherapy adverse events (neuropathy from vinca alkaloids, platinum agents) or paraneoplatic causes [12]. Neurological paraneoplastic syndromes that have been reported with HL include limbic encephalitis, subacute myelopathy, subacute motor neuropathy, GBS, central pontine myelinolysis, and diffuse cerebritis [13].

GBS is described as an inflammatory immune-regulated neuropathy [12]. Incidence is rare, occurring in one to two in 100,000 people worldwide [4] and it is thought to be related to production of antibodies that can be triggered by infectious agents such as C*ampylobacter jejuni,* cytomegalovirus, Epstein-Barr virus, *Mycoplasma pneumonia, Haemophilus influenza,* and variacella-zoster. However, ganglioside and glycolipid targets for the antibodies exist not only in the invading pathogen’s proteins, but also in the tissues of the nervous system. The resulting autoimmune response causes demyelination manifesting as areflexia and ascending motor neuropathy [2]. Similarly, antigens present on the surface of lymphoma cells can also generate production of antibodies that will attack similar proteins in the nervous system [1, 12].

A standard chemotherapy regimen for first-line treatment of classical HL is the four-drug combination containing doxorubicin, bleomycin, vinblastine and dacarbazine (ABVD). Vinblastine can induce numbness and peripheral neuropathy, and patients with preexisting neurologic disorders may be at higher risk of developing neuropathies from vinblastine [14]. This presents a difficult challenge in patients with HL experiencing GBS. In this case, our patient received full-dose vinblastine for four out of 10 doses, and a reduced dose for three doses.

The neurologic abnormalities of GBS can also manifest as respiratory symptoms, seen in 25% of cases [15]. This can complicate decisions to include bleomycin in the treatment regimen, a drug that has resulted in pulmonary interstitial infiltrates in 3 to 5% of patients [14]. Our patient received full doses of bleomycin for seven out of 10 doses, but it was withheld for three doses due to a decline in respiratory status. Certainly, the mechanism of bleomycin-induced lung injury differs from that seen in a neurologic condition such as GBS, but the presence of GBS does make it more difficult to manage a patient like this in an effort to maximally treat the patient without causing underlying damage from chemotherapy agents.

## Conclusions

Given the rarity of these syndromes, it is not completely known what effect, if any, paraneoplastic syndromes have on the overall prognosis of patients with HL. In this case, adjustments made to our patient’s initial chemotherapy regimen may have decreased the effectiveness of the treatment; or the use of immunosuppressive agents such as immune globulin may have potentially stimulated growth of the lymphoma [1]. Since these treatment effects are unknown, it would be valuable to complete a comprehensive case series to estimate the impact of paraneoplastic syndromes and GBS on outcomes in patients with HL to help guide future treatment.

## Consent

Written informed consent was obtained from the patient for publication of this case report and any accompanying images. A copy of the written consent is available for review by the Editor-in-Chief of this journal.

## Abbreviations

ABVD: adriamycin, bleomycin, vinblastine, dacarbazine
AIDP: acute inflammatory demyelinating polyneuropathy
DLCO: carbon monoxide diffusing capacity
ECOG: Eastern Cooperative Oncology Group
GBS: Guillian-Barré syndrome
Gy: Gray
HL: Hodgkin lymphoma
ICE: ifosfamide, carboplatin, etoposide
IMRT: intensity-modulated radiation therapy
IVIG: intravenous immune globulin
MRI: magnetic resonance imaging
NHL: non-Hodgkin lymphoma
PET/CT: positron emission tomography/computed tomography
PFTs: pulmonary function tests.
